# Study on the Effect of Phillyrin on *Streptococcus suis* In Vivo and In Vitro

**DOI:** 10.3390/biom14121542

**Published:** 2024-12-01

**Authors:** Fangyan Yuan, Lihan Zheng, Mengzhe Wang, Wei Liu, Xiaoyue Li, Ting Gao, Rui Guo, Zewen Liu, Keli Yang, Chang Li, Qiong Wu, Jiajia Zhu, Yongxiang Tian, Danna Zhou

**Affiliations:** 1 Key Laboratory of Prevention and Control Agents for Animal Bacteriosis (Ministry of Agriculture and Rural Affairs), Hubei Provincial Key Laboratory of Animal Pathogenic Microbiology, Institute of Animal Husbandry and Veterinary, Hubei Academy of Agricultural Sciences, Wuhan 430064, China; fyyuan@hbaas.com (F.Y.);; 2 State Key Laboratory of Agricultural Microbiology, College of Veterinary Medicine, Cooperative Innovation Center for Sustainable Pig Production, Huazhong Agricultural University, Wuhan 430070, China; zhenglihan2022@163.com (L.Z.);

**Keywords:** phillyrin, *S. suis*, action mechanism, cell adhesion, biofilm

## Abstract

As a zoonotic pathogen, *S. suis* serotype 2 (SS2) can cause severe diseases in both pigs and humans, and develop resistance to antibiotics. Plant natural compounds are regarded as promising alternatives to conventional antibiotics. Phillyrin is the major bioactive components of Chinese herbal medicine Forsythia suspensa. In this study, we explored the activity and action mechanism of phillyrin against SS2. The results showed that phillyrin could disrupt membrane integrity, destroy intracellular structures, and increase the exosmosis of DNA. Results of PCR revealed that phillyrin affected bacterial-virulence-related genes’ expression levels. Meanwhile, phillyrin significantly decreased the adhesion activity, inhibited lactate dehydrogenase (LDH) secretion, and reduced biofilm formation of SS2 in Newborn pig trachea epithelial (NPTr) cells. Furthermore, phillyrin protected tight junction protein of NPTr cells from SS2. We reported that phillyrin (0.1 mg/kg) treatment after bacterial challenge significantly improved the survival rate, ameliorated pulmonary inflammation, and inhibited the accumulation of multiple cytokines (IL-1, IL-6, IL-8, and TNF-α). Molecular docking showed that phillyrin had a good binding activity with the Ala88 and Asp111 of suilysin (SLY), one of the most important virulence factors of SS2. Collectively, phillyrin possesses antibacterial and anti-inflammatory activities, and is a promising candidate for preventing SS2 infection.

## 1. Introduction

*Streptococcus suis* (*S. suis*) is an encapsulated Gram-positive bacterium and mainly colonizes in the upper respiratory tract [1]. Based on capsular polysaccharides antigens, *S. suis* is classified into 29 serotypes [2]. It was reported that serotypes 1, 2, 5, 9, 14, 16, 28, and 31 can infect humans [3,4,5,6]. Among all serotypes, *S. suis* serotype 2 (SS2) is the most highly pathogenic and epidemic strain [7,8,9]. As a zoonotic pathogen, SS2 cause a wide range of clinical disease syndromes including arthritis, meningitis, pneumonia, septicaemia, endocarditis, polyserositis, abortions, and abscesses in swines, but also cause meningitis, septicaemia, endocarditis, and streptococcal toxic shock-like syndrome in humans [10,11]. Since the first human case of SS2-induced meningitis was reported in 1968, more than 1600 cases have been reported with intensive pig production worldwide [12]. Furthermore, SS2 was the major causes of adult meningitis in Hong Kong, Vietnam, and Thailand, respectively [13,14,15]. Two isolates of SS2 potentially hazardous to humans, and some other serotypes, were obtained from 19 out of 47 samples by combining LAMP with a replica plating method [16]. SS2 was shown to be hazardous to humans and regarded as a new zoonotic food-borne pathogen [17,18].

Vaccination is an important tool to prevent infections. Although there were inactivated vaccines and subunit vaccines commercially, no efficacious vaccine is available to prevent all kinds of *S. suis* infections because of the high genomic diversity between strains and the presence of multiple serotypes [19,20,21,22,23]. Antibiotic therapy was the common way to control *S. suis* infection. Multiple classes of antibiotics, including β-lactams, aminoglycosides, amphenicols, sulfonamides, and fuoroquinolones, were used to treat *S. suis* infection [24,25]. On the other hand, the abuse of antibacterial drugs has caused severe antimicrobial resistance (AMR) [26,27]. However, *S. suis* show AMR and multidrug resistance (MDR), closely followed by the widespread and irrational use of antibacterial drugs. For example, 86% of *S. suis* collected from France between 1994 and 2020 were resistant to antibiotics. The resistance percentages of tetracycline, clindamycin, lincomycin, erythromycin, tilmicosin, and tylosin were 81% (*n* = 162), 71% (*n* = 142), 71% (*n* = 142), 69% (*n* = 137), 69% (*n* = 137), and 69% (*n* = 138), respectively [28]. During 246 strains of *S. suis* isolated from diseased pigs in Thailand from 2018 to 2020, the ratio drug resistance rate of more than 90% was seen in clindamycin (99.6%), tetracycline (99.2%), tilmicosin (98.0%), tylosin tartrate (98.0%), rythromycin(97.2%), azithromycin (96.1%), oxytetracycline (96.3%), and chlortetracycline (95.5%) [9]. *S. suis* isolates from humans and pigs in China were resistant to amikacin and tetracycline, and showed high frequencies of resistance to lincomycin (96.9%), erythromycin (91.7%), azithromycin (91.7%), clindamycin (91.7%), and tilmicosin (90.6%). Less than 20% of S. suis strains isolated from Jiangxi province from 2017 to 2019 were susceptible to vancomycin, penicillin, minocycline, and chloramphenicol [29]. Therefore, there is an urgent need to find nonantibiotic and efficient natural compounds to prevent and treat *S. suis* and provide a new strategy for controlling *S. suis* in clinical practice.

Natural compounds are regarded as promising alternatives to conventional antibiotics. Phillyrin is the major polyphenolic compound of Forsythia suspensa (Thunb.) Vahl, also known as Lianqiao in Chinese traditional medicine, has exerted anti-inflammatory, sterilization, and anti-allergy functions, suppressing pathogen virulence factors, and alleviating oxidative stress and inflammation [30,31,32,33,34]. However, the effects of phillyrin on SS2 and the mechanism of action still remain unknown. In this study, the researchers evaluated the bacteriostatic effect of phillyrin on SS2 strain SC19 using the broth microdilution method. Then, we examined the bacterial integrity by scanning electron microscopy (SEM) and transmission electron microscopy (TEM). Additionally, a series of biological activity including biofilm formation, LDH, and cytotoxicity in NPTr cells were studied. Finally, we evaluated the effect of phillyrin on SC19 in mice. Overall, this study contributes to a deeper understanding of the therapeutic targets and pathogenesis of SS2 infection.

## 2. Materials and Methods

### 2.1. Cell Culture, Bacterial Strains, and Compounds

NPTr cells were preserved by our laboratory and cultured in Dulbecco’s modified eagle medium (DMEM, Cytiva, Washington, DC, USA) supplemented with 10% inactivated fetal bovine serum (Gibco, New York, NY, USA), 100 U/mL penicillin, and 100 U/mL streptomycin (Solarbio, Beijing, China) at 37 °C containing 5% CO_2_. The SS2 strain SC19 was grown in tryptic soy broth (BD, Rockville, MD, USA) medium or on tryptic soy agar (BD, Rockville, MD, USA) plates, with 10% inactivated newborn bovine serum (Sijiqing, Hangzhou, China) at a temperature of 37 °C. Phillyrin had the following components: Must, Chengdu, China; CAS number: 487-41-2; Molecular formula, C27H34O11; molecular weight is 534.56. During curve-killing tests, SS2 SC19 was cultured in Mueller–Hinton broth (BD, Rockville, MD, USA) or on MHB agar (BD, Rockville, MD, USA) at 37 °C.

### 2.2. Susceptibility Testing and Growth Characteristic Analysis

The minimum inhibitory concentration (MIC) and minimum bactericidal concentration (MBC) of phillyrin was determined by the standard CLSI guidelines [35]. The serial two-fold dilution method was applied to 96-well micro-titer plates. The SC19 was adjusted to final concentration of 5 × 10^5^ CFU/mL. Bacterial liquid and phillyrin were added to 96-well plates at a ratio of 1:1. The final concentrations of phillyrin were 4096, 2048, 1024, 512, 256, 128, 64, 32, 16, and 8 μg/mL. The turbidity was observed after incubation at 37 °C for 12 h.

The time-killing curve of phillyrin against SC19 is carried out roughly as follows: bacteria SC19 cultured overnight was subcultured with phillyrin at MIC concentrations. Bacteria growth curves were measured by OD600 nm (Victor Nivo, PerkinElmer, Waltham, MA, USA) and CFU were counted every hour for 24 h at 37 °C. The bacterial without phillyrin was used as a control.

### 2.3. Electron Microscope Observation of Bacterial Integrity

TEM and SEM were used to test the effect of phillyrin on the bacterial integrity [36]. SC19 grown in TSB were co-incubated with phillyrin of MBC concentration for 6 h, and then fixed with 2.5% electron microscope glutaraldehyde at 4 °C overnight. Following this, the bacterial cells were treated with 1% osmium tetroxide for 2 h and dehydrated using ethanol and acetone. Therefore, the dehydrated sections were embedded in epoxy resin and dyed with uranium and led double staining. Finally, the cell morphology was observed by a TEM (HITACHI, HT7700, Tokyo, Japan), and the dried cells were analyzed via a SEM (HITACHI, SU8100, Tokyo, Japan).

### 2.4. DNA Exosmosis Determination

The determination of DNA exosmosis was conducted as reference with minor modification [37]. The SC19 solution was washed with PBS and prepared for bacterial suspension (1 × 10^7^ CFU/mL). Phillyrin was added at different concentrations (0, 64, 128 μg/mL) to test wells, respectively. Bacteria with the same volume of DMSO were set as controls. After being incubated at 37 °C for 0, 1, 2, 4, 6, and 8 h, the bacteria were collected, respectively, by centrifugation. The DNA in supernatant was determined using a microspectrophotometer Nano Drop One (Thermo Scientific, Massachusetts, MA, USA).

### 2.5. Biofilm Formation Detection Assay

The biofilm formation assay was conducted as described previously with minor modifications [38]. Phillyrin was added to the SC19 cultured in TSB at MIC and MBC, respectively, in 24-well polystyrene plates for 48 h at 37 °C. TSB without phillyrin was used as a negative control. The biofilms were washed three times with normal saline and stained with 0.1% crystal violet for 15 min. Subsequently, the biofilms were washed and dried. A 33% acetic acid solution was added, and the plates were measured at OD590 using a multifunctional enzyme reader (Victor Nivo, PerkinElmer, Waltham, MA, USA).

### 2.6. Determination of Hemolytic Activity

Suilysin (SLY) contributes to the infection and cytotoxicity of bacteria. Hemolytic activity assay was used to evaluate the effect of phillyrin on SS2. Briefly, phillyrin was added to SC19 at stage of logarithmic growth at 1/4 MIC, 1/2 MIC, MIC, 2 MIC, and, 4 MIC, respectively, and then were placed in a constant temperature incubator at 37 °C and incubated; the same volume of DMSO was used as the untreated group. After incubation for 30 min, 25 μL defibrillated sheep blood was added to each group and incubated in a constant temperature incubator at 37 °C for 30 min. Finally, the co-incubated culture was centrifuged (10,000 r/min, 2 min), and the absorbance value (OD540 nm) of supernatant was detected using a spectrophotometer (Victor Nivo, PerkinElmer, Waltham, MA, USA).

### 2.7. Calcein/PI Cell Activity Assay

The protective effect of phillyrin on SC19-infected NPTr cells was explored by Calcein AM/PI staining. The operation procedure has been slightly modified according to the references [39]. Briefly, the NPTr cells were cultured in DMEM and cultured overnight at 37 °C. Following incubation, the cells were washed gently with sterile PBS three times. The cells were infected with SC19 at a multiplicity of infection (MOI) of 100 and incubated for 2 h. Subsequently, after washing with PBS, phillyrin was added to the cells at MIC and 2 MIC, respectively, and then incubated for 6 h, with fresh DMEM without phillyrin employed as a negative control. Following this, the mixes were cleaned with PBS, and 500 μL Calcein AM/PI dyeing solution was added per well. Finally, acting for 15–20 min at room temperature without light, the labeled cells were observed under a fluorescence microscope. Visualization was performed using a Live/Dead Cell Double Staining Kit (Proteintech Group, Wuhan, China).

### 2.8. Cytotoxicity Assay

The cytotoxicity of phillyrin was detected by CCK-8 assay. NPTr cells cultured overnight were washed three times with PBS. Phillyrin at final concentration (0 MIC, 1 MIC, 2 MIC, and 4 MIC) was added to each test well, respectively. Only cells and only culture medium were set as control wells and blank wells, respectively. After 6 h incubation, 10 μL CCK-8 solution (MedChemexpress, New Jersey, USA) was added to each well. The absorbance of the supernatant at 450 nm was measured using an EnSight^®^ Multimode Plate Reader after co-incubation for 2 h. The following formula was used to evaluate the cell viability, Equation (1): Cytotoxicity = (test well absorbance − blank well absorbance)/(control well absorbance-blank well absorbance). The experimental data were calculated using GraphPad Prism 8.

### 2.9. Live/Dead Cell Staining Test

Double fluorescence staining of cells was performed using Live/Dead cell staining kits to detect whether phillyrin protected NPTr cells from SS2. The NPTr cells were seeded into 24-well tissue culture plates and cultured overnight until they reached 100% confluence. The cells were then washed with sterile PBS and infected with SC19 at a multiplicity of infection (MOI) of 100. Next, the culture plates were incubated for 2 h at 37 °C. Subsequently, the culture plates were washed with sterile PBS and cell medium containing 1 MIC, and 2 MIC phillyrin were added, respectively. After incubation for 6 h at 37 °C, the cells were washed and the Calcein AM (2 μM) and Propidium Iodide (PI, 4.5 μM) dyeing solution was lucifugously added to 500 μL per well. Following this, the mixture was incubated at room temperature without light for 20 min. Finally, the labeled cells were observed under a fluorescence microscope.

### 2.10. LDH Detection Assay

The cytotoxicity of phillyrin was detected by LDH assay kit (Nanjing Jiancheng, Nanjing, China). NPTr cells (1 × 10^6^/well) were seeded into 96-well plates. The cells with added phillyrin at concentrations MIC and 2MIC were treatment groups, and cells without added phillyrin were negative controls. The LDH viability was measured using a microplate reader (Molecular Devices, Sunnyvale, CA, USA) at 490 nm. The LDH activities were expressed according to the following formula, Equation (2): Cell mortality (%) = (Test Sample − Low Control)/(High Control − Low Control) × 100%.

### 2.11. Adherence Assay

NPTr cells were seeded into 24-well culture plates with 108 cells per well at 37 °C in 5% CO_2_ overnight, and then were pretreated with phillyrin (1/4 MIC, 1/2 MIC, 1MIC) for 3 h. Cells without phillyrin were used as a negative control. SC19 was added to NPTr cells at an MOI of 10:1 and co-incubated for 2 h and were washed three times with PBS. The cells attached by bacteria were lysed with 0.025% Triton X-100 (Sinopharm Chemical Reagent Co., Ltd., Shanghai, China) on ice for 15 min. Finally, the bacteria attached to the cells are counted.

### 2.12. Real-Time PCR Analysis

RNA expression of virulent-related genes of SC19 was evaluated to explore the mechanism of the antibacterial activity of phillyrin. SC19 was cultured to the logarithmic metaphase, and phillyrin was added at 64 µg/mL. Co-cultured for 4 h, the total RNA was reverse transcribed using the HiScript II First-Strand cDNA Synthesis Kit (Vazyme, Nanjing, China) in accordance with the protocol. Primers [40,41] were listed in Table 1. The 16SrRNA gene was used as the internal control. Differentially expressed genes were judged by fold change > 2 and q-value < 0.05.

### 2.13. Animal Experiment

Animal studies were conducted according to the animal welfare guidelines of the World Organization for animal health. All animal studies were approved by the Ethics Committee of the Institute of Animal Husbandry and Veterinary, Hubei Academy of Agricultural Sciences (Wuhan, China; identification code: XCXK(E)2020-0018). Five-week-old female specific pathogen-free (SPF) BALB/c mice were purchased from Hubei Experimental Animal Centre (Wuhan, China), and were used to evaluate the protection afforded by phillyrin. After 7 days of adaptation, the mice were randomly divided into 4 groups. All groups were given common feed, and at the same time, group 2 and group 3 were given phillyrin at a dose of 50 mg/kg/d and 100 mg/kg/d by gavage every day, respectively. Group 4 was administered normal saline as a negative control. After continuous gavage for 3 days, all mice were intraperitoneally infected with 3 × 10^9^ CFU/mouse of SC19. The daily clinical symptoms, morbidity, and mortality of the mice were recorded for 7 days.

In the tissue load test, 12 five-week-old female SPF BALB/c mice were randomly divided into 2 groups. Group 1 was given phillyrin at a dose of 100 mg/kg by gavage. After 30 min, Group 1 was intraperitoneally infected with 7 × 10^8^ CFU/mouse of SC19. Group 2 was administered normal saline as a negative control. At 12 h and 24 h, 3 mice in each group were sacrificed, and the brain, lungs, and blood were collected for bacteria counts. In addition, blood was also used to measure IL-1, IL-6, IL-8, and TNF-α using ELISA Kits.

### 2.14. Molecular Docking Assay

The interaction between phillyrin and SLY was analyzed by the molecular operating environment (MOE). The chemical structure of phillyrin was downloaded from the website PubChem “https://pubchem.ncbi.nlm.nih.gov/ (17 April 2024)”. The three-dimensional structure of SLY was obtained from the Protein Database “https://www.wwpdb.org/ (17 April 2024)”.

### 2.15. Statistical Analysis

All experiments were repeated three times and statistically analyzed using GraphPad Prism version 8 (GraphPad Software, San Diego, CA, USA). Results are expressed as the mean ± standard deviation (SD). Statistical significance was analyzed by Student’s t test, and *p* values of <0.05 (*), <0.01 (**), and <0.001 (***), and <0.0001 (****) were considered to indicate different levels of statistical significance.

## 3. Results

### 3.1. Phillyrin Inhibits the Growth of SS2 In Vitro

**Figure 1:**
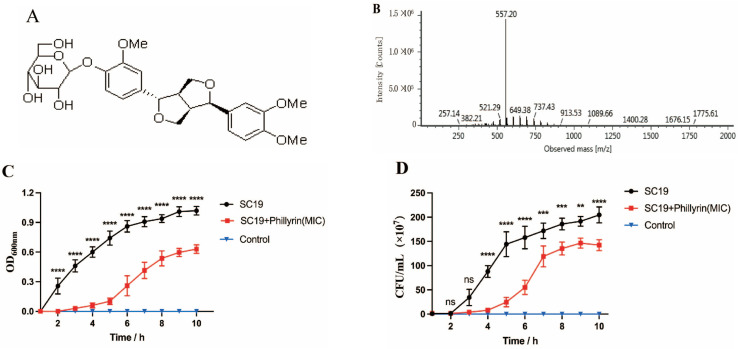
Antibacterial activity of phillyrin against SC19 in vitro. (**A**) Chemical structure of phillyrin. (**B**) Analysis of phillyrin by Mass Spectrum. (**C**) The growth curve of SC19 affected by phillyrin was determined by OD600 nm at the indicated times. (**D**) The growth curve of SC19 affected by phillyrin was determined by CFU counts at the indicated times. (ns, *p* > 0.05; ** *p* < 0.01; *** *p* < 0.001; **** *p*< 0.0001).

Phillyrin was extracted from fructus forsythiae and the purity was 99.09% (Figure 1A,B). The MIC and the MBC of phillyrin against SC19 was 64 µg/mL and 512 µg/mL by the micro-broth dilution assay, respectively (Table 2).

To evaluate the effect of phillyrin on proliferation of SS2, we co-cultured SC19 with phillyrin at the concentrations of 0 µg/mL, 8 µg/mL (1/8 MIC), 16 µg/mL (1/4 MIC), 32 µg/mL (1/2 MIC), 64 µg/mL (1 MIC), and 128 µg/mL (2 MIC). After being co-incubated for 6 h, the OD600 nm value of SC19 treated by phillyrin was only about 0.3; meanwhile it was 0.8 in the untreated bacteria. And in the growth curve, the amount of SC19 was 3.2-fold less than that of the untreated SC19 (*p* < 0.001) (Figure 1C,D). The results showed that phillyrin significantly inhibited the growth of SC19 in the stationary phase.

**Figure 2:**
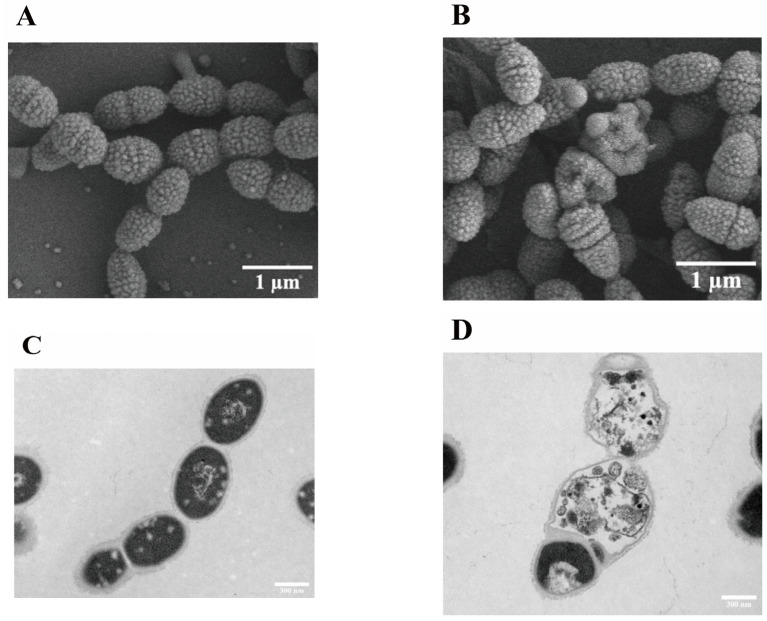
Scanning electron microscopy (SEM) and transmission electron microscopy (TEM) of SS2 after treatment with phillyrin. (**A**) TEM observation of phillyrin-untreated SC19; the bar at the bottom right means 1 µm. (**B**) TEM observation of phillyrin-treated SC19; the bar at the bottom right means 1 µm. (**C**) SEM observation of phillyrin-untreated SC19; the bar at the bottom right means 300 nm. (**D**) SEM observation of phillyrin-treated SC19; the bar at the bottom right means 300 nm. Bacterial cell presented shrinkage, cell size reduction, and perforation of the cell surface. Control cells without treatment appeared with normal shape.

The effect of phillyrin on the cell integrity of SC19 was directly visualized by a TEM and a SEM. Comparing with the smooth surface, clear edge, and complete cell wall and membrane of non-medicated SC19 (Figure 2A,C), the bacteria treated by phillyrin at MBC showed instances of cell wall damage and ruptured membranes, accompanied by the leakage of cytoplasmic contents (Figure 2B,D).

### 3.2. Phillyrin Increases DNA Exosmosis of SS2

**Figure 3:**
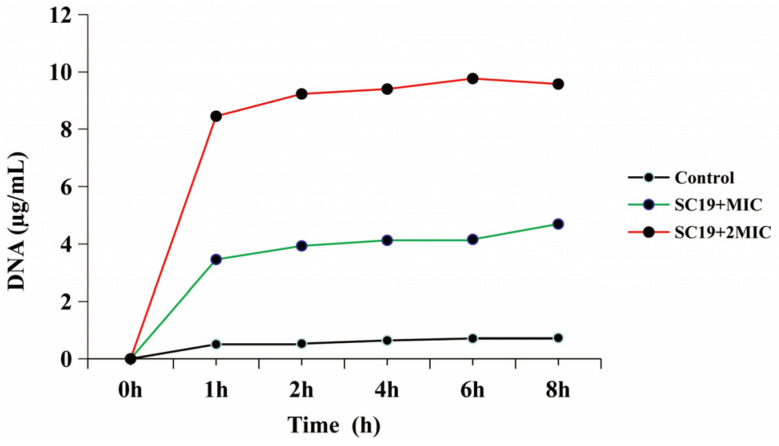
Phillyrin signifcantly improves the DNA exosmosis of SS2. SC19 was cultured to the logarithmic stage, blended to 107 CFU/mL and treated with phillyrin at 64 µg/mL and 128 µg/mL respectively.SC19 without phillyrin were used as a negative control. Supernatants were collected after co-incubated for 0, 1, 2, 4, 6 and 8 h, and the DNA content was determinated with a microspectrophotometer.

To verify the potential effects of phillyrin on cell membranes and cell walls, a DNA exosmosis assay was performed. The bacteria were co-incubated with phillyrin (1MIC, 2MIC), and SC19 without phillyrin was used as control group. The samples were cultured for 0, 1, 2, 4, 6 and 8 h and the DNA content was determined with a microspectrophotometer. As shown in Figure 3, compared with the control group, after adding phillyrin for 8 h, the DNA exosmosis of SC19 with MIC and 2MIC increased to (7.21 ± 0.63) µg/mL and (14.08 ± 0.19) µg/mL (*p* < 0.01), respectively. The results indicated that phillyrin could significantly improves the amount of DNA exosmosis.

### 3.3. Phillyrin Inhibits the Secretion of Hemolysin from SS2 Dose-Dependently

**Figure 4:**
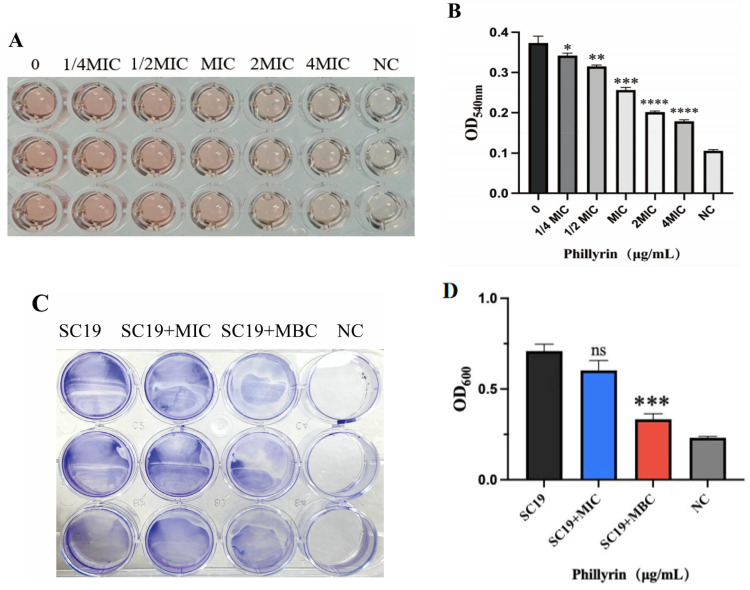
Phillyrin inhibits the secretion of hemolysin and biofilm information of SS2. (**A**,**B**) Hemolytic activity analysis of SC19 affected by PHI. SC19 was cultured to the logarithmic stage, treated with different concentrations (1/4 MIC, 1/2 MIC, MIC, 1/4 MBC and 1/2 MBC) of phillyrin. After incubation with defibrillated sheep blood, the hemolysin was collected and detected by a spectrophotometer. (**C**,**D**) Biofilm formation analysis of SC19 affected by phillyrin. The biofilms of the SC19 with phillyrin at MIC and MBC (512 µg/mL) were stained by the crystal violet method and OD600 was determined after it dissolved. ns, *p*>0.05; * *p* < 0.05; ** *p* < 0.01; *** *p* < 0.001; **** *p* < 0.0001 compared to the respective control.

To verify the inhibitory effect of phillyrin on SS2, the hemolysin of SC19 was measured. SC19 was treated with different concentrations (1/4 MIC, 1/2 MIC, 1 MIC, 1/4 MBC and 1/2 MBC) of phillyrin. The bacteria were incubated with defibrillated sheep blood for 30 min. Then, the hemolysin was collected and detected by a spectrophotometer. The mean absorbance value (OD540 nm) of SC19 treated with different concentrations (0MIC, 1/4 MIC, 1/2 MIC, 1 MIC, 2MIC, and 4 MIC) of phillyrin was 0.37, 0.34, 0.32, 0.26, 0.20, and 0.18, respectively. The results showed that phillyrin significantly reduced the secretion of hemolysin in a dose-dependent manner (Figure 4A,B).

Biofilm formation plays a critical role in natural and anthropogenic environments. In order to determine the effect of phillyrin on the formation ability of SS2 biofilm, biofilm formation was stained by the crystal violet method and OD600 was determined after it dissolved. The average absorbance value of SC19 treated with different concentrations (0MIC, MIC, MBC) of phillyrin was 0.71, 0.60, and 0.33. Compared with the bacteria without phillyrin, the biofilms of the SC19 with phillyrin at MBCs were significantly inhibited (kp < 0.001), but there was no significant difference at MIC (*p* > 0.05) (Figure 4C,D). Our results demonstrated that phillyrin was effective in inhibiting biofilm formation of SS2.

### 3.4. Phillyrin Has a Protective Effect and No Cytotoxicity on NPTr Cells

**Figure 5:**
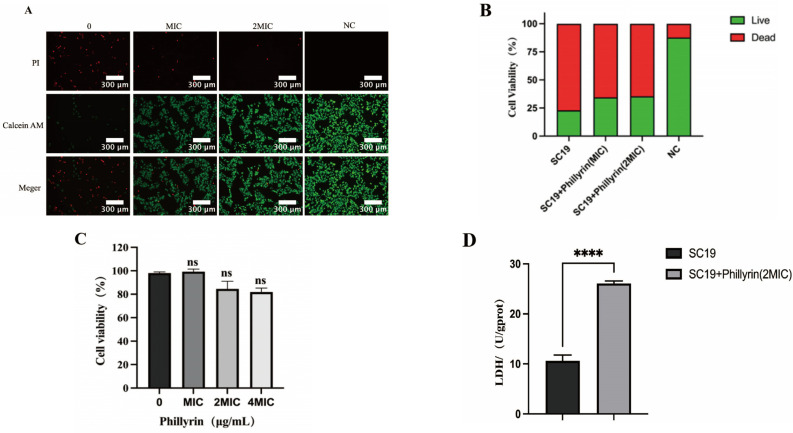
Effect of phillyrin on NPTr cells. (**A**) Analysis of cytotoxicity of phillyrin by double fluorescence staining of NPTr cells. (**B**) Cell viability was determined by a CCK8 assay. (**C**) Analysis of the cytotoxic activity of phillyrin by standard LDH release assay. ns, *p* > 0.05. (**D**) Results were expressed as the percentage of LDH release compared to the non-phillyrin cells. Error bars represent the standard deviation of three independent experiments performed in triplicate. **** *p* < 0.0001.

To evaluate the potential effects of phillyrin on the cytotoxicity in NPTr cells, CCK-8, which is a highly sensitive and non-radioactive colorimetric method, was used to determine the number of living cells. The cytotoxic activity of NPTr cells pretreated with phillyrin at 2 MIC and 4 MIC had a slight but not significant decline, compared with the control group (*p* < 0.05) (Figure 5A,B). The result proved that the phillyrin has no cytotoxicity in NPTr cells.

To verify the protective effect of phillyrin on SS2, NPTr cells were infected with SC19 and treated with different concentrations of phillyrin (1MIC, 2MIC). The cell viability was detected by a Calcein AM/PI double probe. Living cells showed green fluorescence, and dead cells showed red under fluorescence microscope. The results are shown in Figure 5C; compared with the untreated group, the red fluorescence of NPTr cells decreased after being treated by phillyrin, while the green fluorescence increased. These results indicated that phillyrin had a certain protective effect on SC19-infected cells, and 2 MIC phillyrin had a greater protective effect on cells than MIC.

Lactate dehydrogenase (LDH) is a cytoplasmic enzyme secreted from different types of cells and is recognized as a reliable indicator of cytotoxicity. After co-culture of SC19 with phillyrin at 2MIC (128 μg/mL) for 5 h, the LDH content in the bacteria was as high as 26.08 U/gprot, which was significantly higher than that of SC19 without phillyrin. Our results indicated that phillyrin could increase the LDH secretion in SC19 (Figure 5D).

### 3.5. Phillyrin Reduces the Adherence Ability of SS2 to NPTr Cells

**Figure 6:**
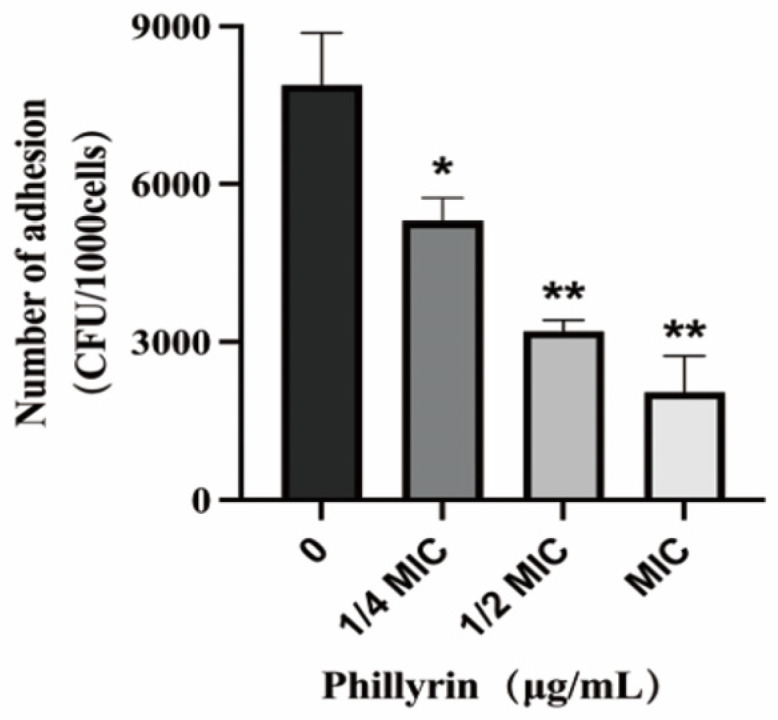
Adhesion ability analysis of SS2 affected by phillyrin. NPTr cells were treated by SC19 with phillyrin, and then the number of adherent bacteria was measured. The experiment was performed in triplicate and repeated at least three times. * *p* < 0.05; ** *p* < 0.01.

In order to study the effect of phillyrin on cell adhesion of SC19, NPTr cells were treated by SC19 with phillyrin at final concentrations of 1/4 MIC, 1/2 MIC, and 1MIC, respectively, and then the number of adherent bacteria was measured after incubation for 2 h. Results As shown in Figure 6, compared with the control group, phillyrin could significantly inhibit the adhesion of SC19 to NPTr cells dose-dependently.

### 3.6. Phillyrin Reduces Damage of Cell Tight Junction Protein by SS2

**Figure 7:**
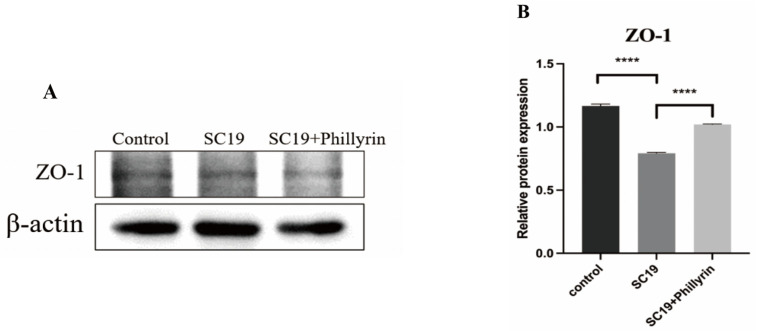
Phillyrin reduces damage of cell tight junction protein by SS2. (**A**) Expression of cell tight junction protein ZO-1 was detected by Western blot. (**B**) Western blot analysis of phillyrin treatment on the tight junction protein ZO-1 of NPTr cells infected by SC19 with or without 64 µg/mL phillyrin. The height of the bars indicates the mean values for the relative expression data ± SEM (**** *p* < 0.0001). Original images of (**A**) can be found in supplementary materials.

**Figure 8:**
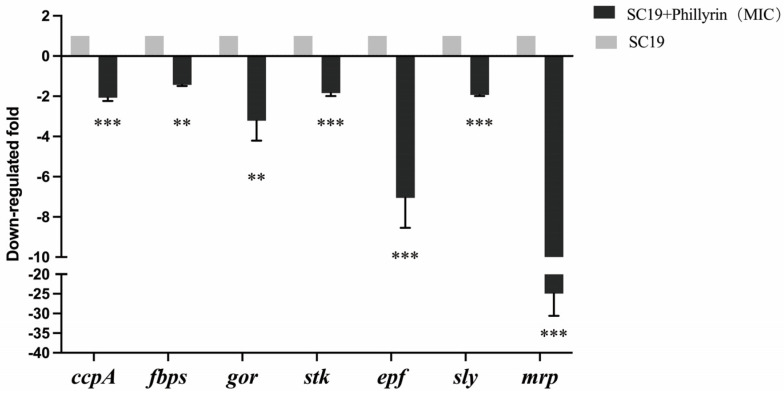
Phillyrin suppresses genes expression of SS2.The real-time PCR results of virulence factor genes (*mrp, epf, sly, stk*), cell adhesion-related genes (*ccpA, fbps*), and cell division-related genes (*gor*) of SC19. ** *p* < 0.01; *** *p* < 0.001 compared to the respective control.

To investigate the effect of phillyrin on the tight junction (TJ) protein of NPTr cells, ZO-1 protein expression infected with SC19 was detected by Western blot. Results are shown in Figure 8; compared with the control group, the contents of ZO-1 pretreated with phillyrin up-regulated significantly (**** *p* < 0.0001). The result indicated that phillyrin can significantly restore the loss of TJ proteins caused by SS2 (Figure 7A,B).

### 3.7. Phillyrin Affected Gene Expression Levels of SS2

To study the effect of phillyrin on gene expression levels of SS2, the virulence factor genes (*mrp*, *epf*, *sly*, *stk*), cell adhesion-related genes (*ccpA*, *fbps*), and cell division-related genes (*gor*) of SC19 was tested by real-time PCR. The results showed that the expression level of all the above genes was decreased after treatment with phillyrin, in which the most significant declines were in *mrp*, *epf* (Figure 8). The result indicated that phillyrin can affected gene expression levels of SS2.

### 3.8. Effect of Phillyrin on Mice Infected by SS2

**Figure 9:**
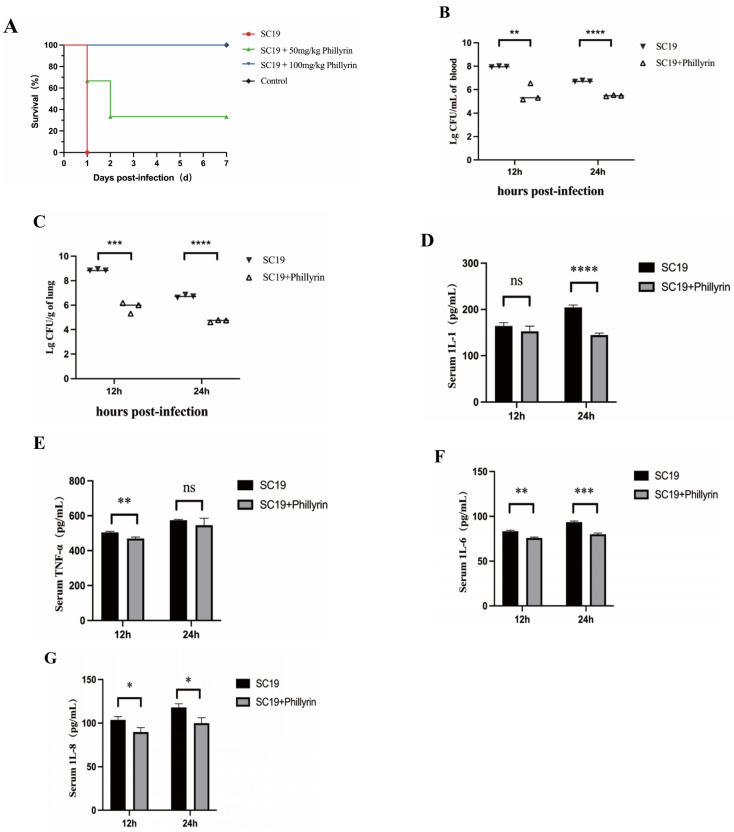
Phillyrin reduced the virulence of SS2 in mice. (**A**) Virulence assay of SC19, SC19+phillyrin, and normal saline by comparing the survival of posttreatment mice. Bacterial load in the blood (**B**) and lung (**C**) tissues of the SC19-infected mice. Secretion of IL-1ß (**D**), IL-6 (**E**), IL-8 (**F**), and TNF-α (**G**) in the serum of mice infected with SC19. The height of the bars indicates the mean values for the relative expression data ± SEM (ns, *p* > 0.05; * *p* < 0.05; ** *p* < 0.01; *** *p* < 0.001; **** *p* < 0.0001).

The protective effect of phillyrin on mice infected by SS2 was evaluated by survival rate and colonization of bacteria in tissues. In the survival experiment, all mice infected with SC19 and without phillyrin treatment showed severe clinical symptoms, such as trembling, paddling, and deranged fur, and died within 24 h. Predictably, administration of phillyrin significantly improved these symptoms. At the same time, the survival rate of phillyrin-treated (50 µg/kg and 100 µg/kg) infected mice increased to 33.33% and 100%, respectively (Figure 9A).

In the colonization of bacteria experiment, mice were infected with 7 × 10^8^ CFU/mL SC19, and were killed at 12 h and 24 h after infection, respectively. The blood and lungs were separated. The number of viable bacteria in the tissues of mice was measured. Results shown that the count of SC19 was higher than that of the phillyrin-treated group at 0.5 and 1 dpi (Figure 9B,C). Furthermore, serum was collected and pro-inflammatory factors IL-1β, IL-6, and TNF-α were detected. Compared with the group given SC19 without phillyrin treatment, the expression levels of IL-6 and TNF-α of phillyrin-treated mice were significantly suppressed at 0.5 dpi (Figure 9E–G), and similar results were seen for IL-1 and IL-6 at 1 dpi (Figure 9D,F,G).

### 3.9. Binding Site of Phillyrin to SLY of SS2 Revealed by Molecular Docking

To understand the action mechanism by which phillyrin inhibits SS2 infection, molecular docking was used to predict the binding sites between phillyrin and SLY. As shown in Table 3, a total of 32 pockets that might bind to small molecules were predicted.

In the highest-scoring pocket (Pocket 1), the optimal binding energy value was −6.1901 kcal/mol (Table 4).

**Figure 10:**
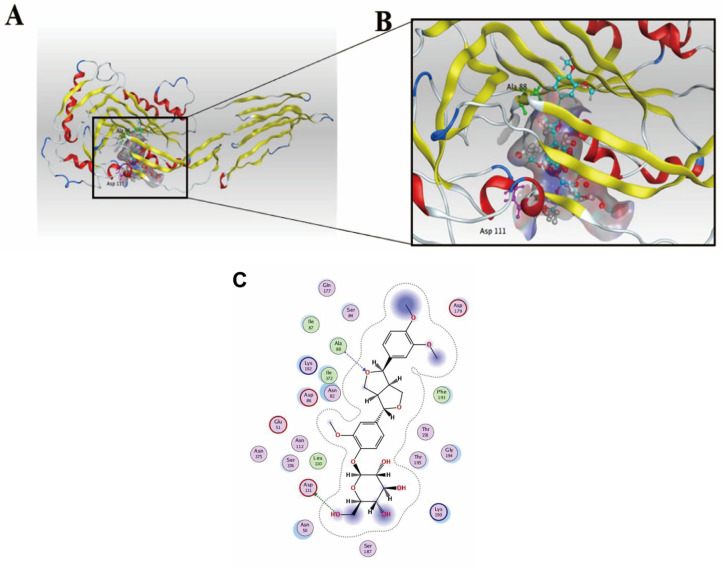
Molecular docking of phillyrin and SLY. (**A**,**B**) Three-dimensional structure of SLY docked with phillyrin, revealing the amino acids Ala88 and Asp111 react with phillyrin. (**C**) Predictive interaction between phillyrin and SLY protein. Two key bonds indicated with the dotted arrows were established.

The three-dimensional structure and the two-dimensional configuration map clearly revealed that phillyrin bonds with amino acids Ala88 and Asp111, respectively, and hydrogen bonding and hydrophobic complementation were probably the main interaction between phillyrin and SLY (Figure 10A–C).

## 4. Discussion

SS2 is a worldwide pathogen causing severe diseases, which caused huge economic losses to the pig industry. In Vietnam, SS2 was reportedly able to infect humans, and occupational exposure and eating contaminated food were considered the primary risk factors. It was reported that the pooled proportions of case-patients with pig-related occupations was 38.1%, and history of eating high-risk food was 37.3% [14]. In Japan, 255 samples tested positive with LAMPSS from 966 raw pork meat samples, and the rate of contamination was higher in the organs than in pork [16]. Therefore, SS2 is also considered a zoonotic food-borne pathogen for being hazardous to humans, and a threat to food safety needs to be recognized [16,42].

The prevention and control of *S. suis* infection has become a problem that must be solved in the porcine industry. Vaccine use is limited by the difficulty of covering multiple serotypes of *S. suis* infections. Antibiotic therapy was helpful but should be used with caution because of drug resistance, and the development of new therapeutic agents is essential. Plant natural flavonoids work against multidrug resistant pathogens due to advantages in accessibility, structural diversity, robust activity, and distinct modes of action [43,44,45,46].

Phillyrin, a furofuran lignan derived from the traditional Chinese plant Forsythia suspensa (Thunb.) Vahl, possesses many pharmacological effects, including anti-inflammatory, antibacterial, antioxidant, antivirus, anti-cancer, and anti-allergy effects, etc. [47]. Jiao et al. also showed that the essential oil of Forsythiae Fructus (MIC = 0.78–6.25 mg/mL) was also effective in resisting all the susceptible microorganisms in vitro, involving *Staphylococcus aureu*, *Bacillus subtilim*, *Escherichia coli*, *Pseudomonas aeruginosa*, *Candida albicans,* and *Aspergillus niger* [48]. Han et al. examined the bacteriostatic activities of an 80% methanol extract of Forsythiae Fructus against *Staphylococcus aureus* (MIC = 12.50 mg/mL) in vitro [49]. Phillyrin exerted anti-inflammatory effects on microglia by mitigating BMEC injury and integrity violation, and promoting the “M2” polarization of microglia [50]. However, the efficacy of phillyrin on SS2 has not been reported. In this present study, we conducted both in vivo and in vitro experiments to evaluate the antibacterial effects of phillyrin on SS2. Our data suggested that the MIC and MBC of phillyrin against SC19 was 64 µg/mL and 512 µg/mL, respectively.

The integrity of the cell wall is the basic condition of normal life activities. The cell membrane is the natural barrier that protects bacteria by blocking the entry of harmful substances such as drugs, toxins, and degrading enzymes, as well as allowing nutrients to enter the cell. Many natural compounds have been reported to have related functions. Tea polyphenols significantly reduced the formation of *Haemophilus parasuis* biofilms at concentrations of 80, 160, and 320 µg/mL relative to the control in a dose-dependent manner [51]. Our research discovered tea polyphenols can influence the formation of *Actinobacillus pleuropneumoniae* and SS2 as well [52,53]. In the present study, the results of TEM and SEM showed that the cell of SC19 when interacted with phillyrin was crumped, the cell wall and membrane were damaged, the cytoplasm was lost, and the inner cavity showed cavitation (Figure 2). Otherwise, we measured DNA exosmosis using a microspectrophotometer Nano Drop One, and discovered that the cell wall and cell membrane of SC19 could be damaged by phillyrin (Figure 3), but the detailed mechanism involved needs further study.

Bacterial biofilm is one of the most important reasons for chronic and persistent infection [54]. A variety of studies had confirmed that some drugs’ resistance mechanisms are associated with the antibiotic resistance of biofilm information. Exposure to sub-MICs of amoxicillin, lincomycin, and oxytetracycline contributes to increased biofilm formation of *S. suis* [55]. There were remarkable reductions in biofilm formation (52.81, 74.31, and 84.47%) when *Pseudomonas aeruginosa* cells were treated with increasing concentrations of phillyrin (0.0625–0.25 mg/mL) [32]. Our study found that phillyrin at MBC (512 µg/mL) can significantly inhibit the biofilms of SC19, but the mechanisms need further study.

In addition to that, the protective effect of phillyrin was proved by cell infection and animal experiments. Phillyrin significantly decreased the adhesion ability of SC19, reduced cell damage, increased the expression of tight junction protein, improved the survival rate of mice and reduced the production of inflammatory cytokines. These results demonstrated that phillyrin had protective effects on the physical and immune barriers of the lung.

## 5. Conclusions

In summary, this study demonstrates that phillyrin at MIC can inhibit the growth and biofilm formation of SS2 in vitro. Phillyrin also reduced the ability of SS2 to adhere to NPTr cells, and promoted the expression of junction proteins to conserve the trachea epithelial barrier integrity. Many genes associated with virulence and cell division were down-regulated after phillyrin treatment. Although we observed this phenomenon, some important questions remain to be resolved. For instance, the possible molecular mechanism by which phillyrin destroys the cellular morphology of SS2. Although significant work remains to be conducted, our findings suggest that phillyrin might be a promising candidate for the prevention and treatment of SS2 infections.

## Figures and Tables

**Table 1:** Primers used for qRT-PCR in this study.

Primers	Primers Sequence	Target Gene	Reference or Source
16s RNA-F	GTTGCGAACGGGTGAGTAA	*16s RNA*	[40]
16s RNA-R	TCTCAGGTCGGCTATGTATCG		
ccpA-F	CGGTGTCAGTGATATGGG	*ccpa*	[40]
ccpA-R	GTCAGGTTTGGACGGGTA		
fbps-F	AACCATCTTGCCAGGCTCCAC	*fbps*	[40]
fbps-R	CAGTTCAGAAGCCGTATCCCGAC		
gor-F	GTTCACGCGCATCCTACG	*gor*	[40]
gor-R	TACCAGGAATAGCAGGGAC		
stk-F	ATGATTCAAATCGGTAAGATCTTTGC	*stk*	This work
stk-R	GCCATTGACATATTCCATAGCC		
epf-F	ACAGATCCAGATAGCGACATTCAAA	*epf*	This work
epf-R	TATTACCAGTGGCATCCGTCAC		
mrp-F	CAGATGTGGACCGTAGACC	*mrp*	This work
mrp-R	CCTTGCCTTCAAAAGTGATGGA		
sly-F	GCTTGACTTACGAGCCACAA	*suilysin*	[41]
sly-R	CCGCGCAATACTGATAAGC		

**Table 2:** Determination of the antibacterial activity of phillyrin on SC19.

Compound	Bacterial Strain	MIC (μg/mL)	MBC (μg/mL)
phillyrin	SC19	64	512

**Table 3:** Predicting the location of pockets where SLY may bind to phillyrin using MOE.

Site	Size	PLB	Hyd	Side	Residues
1	69	3.38	16	40	1:(ANS50 GLU51 GLY52 ASN82 ASN83 SER84 ASP86 ILE87 GLN107 LEU109 LEU110 ASP111 ASN112 SER187 LYS190 THR191 LYS192 PHE193 GLY194 THR195 ILE372 LEU373 SER374 ASN375 SER376)
2	36	2.21	20	30	1:(TYR54 ILE55 TYR184 SER185 MET186 SER187 PHE208 APS209 VAL211 ASN212 GLU377 TYR378 ILE379 THR381)
3	55	1.41	14	38	1:(MET182 PYR184 VAL211 GLU214 GLU215 LYS216 GLN217 SER281 SER282 ARG283 SER284 THR285 GLN286 VAL287 GLN288 ALA289 GLU380 THR381 THR382 SER383)
4	21	1.18	10	17	1:(THR62 GLU63 LEU65 PHE70 GLU409 VEL410 SER411 TYR412 VEL419 GLU421 ASN445)
5	57	0.97	25	36	1:(ILE97 TYR98 PRO99 ARG147 VAL150 ARG151 VAL154 ASN155 LEU158 TYR228 TYR270 GLY325 GLY354 VAL355 PRO356)
6	11	0.84	8	27	1:(PHE70 VAL72 ARG74 THR384 HIS36 TYR412 GLU418 ARG447)
7	43	0.77	16	36	1:(ASN82 SER84 ASP86 ILE87 ALA88 ILE90 ASN112 GLM177 ASP179 LYS192 PHE193 6LY194 THR195 SER196 ASN22 L′S24 PHE275)
8	27	0.60	13	23	1:(ARG272 MET274 ILE319 GLY322 ASP323 LYS340 ILE341 GLU344 GLY345 ALA346 TYR348 GLY349)
9	51	0.39	21	37	1:(VAL89 ILE90 ASP91 ALA94 ALA95 ILE97 ASP111 ASN112 ASN113 LYS190 THR195 SER196 GLU198 LYS199 TYR359)
10	22	0.22	10	14	1:(LEU128 ASN129 LEU130 PRO131 GLY132 LEU133 ALA134 ASN135GLY136 ASP137 TRP161)
11	32	0.19	11	32	1:(ILE80 GLU180 THR181 MET182 TYR184 GLN188 LYS192 GLN288 TYR378 GLU380)
12	29	−0.09	6	15	1:(HIS386 ASN387 SER388 SER389 ILE442 PRO443 GLY444 ASN445 ALA446 ARG447 LEU474 VAL475 GLY476)
13	19	−0.11	9	23	1:(GLU66 ASN67 ARG69 VAL71 GLY214 GLU215 LYS216 SER281 ARG283 VAL385)
14	11	−0.13	6	18	1:(GLU66 ASN67 ARG69 VAL71 GLY214 GL0215 LYS216 SER281 ARG283 VAL385)
15	56	−0.15	19	32	1:(TYR98 LEU102 LEU116 ILE117 SER118 ILE119 ARG121 PRO145 THR146 ARG147)
16	24	−0.17	18	21	1:(THR285 GLN286 ALA289 ALA290 ILE300 ALA304 GLU305 TYR306 GLN307 ILE309 LEU310)
17	15	−0.20	1	7	1:(GLY122 ASP123 SER239 LYS240 LEU241 PHE242 ALA243 GLU244 GLY245)
18	18	−0.25	9	12	1:(ARG447 ASN448 LEU449 ASP471 LEU472 PRO473 LEU474)
19	28	−0.42	19	23	1:(ACE31 ASP32 ILE33 TYR36 VAL248 GLU249 LEU251 LYS252)
20	26	−0.51	6	19	1:(ASN67 GLY68 ARG69 ASN387 SER388 SER389 ALA390 GLN441 GLY476 GLN477 LEU498)
21	9	−0.62	5	9	1:(GLU46 ILE47 LEU48 THR49 ASP106 GLN107 LEU110)
22	17	−0.62	10	20	1:(GLU175 LEU176 GLN177 LYS224 ILE226 THR229 SER269 SER358)
23	10	−0.64	9	14	1:(ALA293 ALA294 ILE295 GLY297 ASP299 ILE300 SER301 LYS339 ILE342 GLU343)
24	10	−0.66	7	17	1:(ASP205 ILE206 ASN207 VAL218 LYS277 GLU279)
25	12	−0.86	7	14	1:(GLU66 VAL71 LEU73 SER213 GLY214 GLU215 ARG283 SER383)
26	10	−0.88	5	11	1:(GLU51 GLY52 GLU53 ASN375)
27	9	−0.89	3	7	1:(PRO235 GLU236 SER237 PRO238 VAL364LYS365 ASN367)
28	9	−0.99	8	14	1:(GLY136 SER138 LYS160 TRP161 TYR165)
29	10	−0.99	3	7	1:(THR49 ASN50 GLU51)
30	7	−0.99	1	9	1:(LYS93 ALA94 ALA95 ASN96 ASN113 ARG147)
31	8	−0.99	2	2	1:(ALA60 THR61 ALA415 GLY416)
32	8	−0.99	3	9	1:(ASN448 LEU449 HIS450 ASP471)

**Table 4:** Gibbs free energy of phillyrin after molecule docking with Pocket 1 of SLY protein.

	Mol	rseq	mseq	S	E_Score1	E_Refine	E_Score2
1	101712	1	1	−6.1901	−10.8269	6.6277	−6.1901
2	101712	1	1	−5.7576	−10.3733	15.3135	−5.7576
3	101712	1	1	−5.2715	−10.2766	7.9008	−5.2715
4	101712	1	1	−5.1763	−11.7010	2.0349	−5.1763
5	101712	1	1	−5.1015	−11.1251	11.8131	−5.1015

## Data Availability

Data are contained within the article.

## Supplementary Materials

The following supporting information can be downloaded at: https://www.mdpi.com/article/10.3390/biom14121542/s1, Original images of Figure 7A can be found in supplementary materials.
