# Supplementary material for: Study on the Effect of Phillyrin on Streptococcus suis In Vivo and In Vitro
Source: Biomolecules. 2024 Dec 1;14(12):1542. doi: 10.3390/biom14121542 (PMC11673059; doi:10.3390/biom14121542)
Supplement: Supplementary file 1 [file biomolecules-14-01542-s001.zip › biomolecules-3309806-supplementary.pdf]

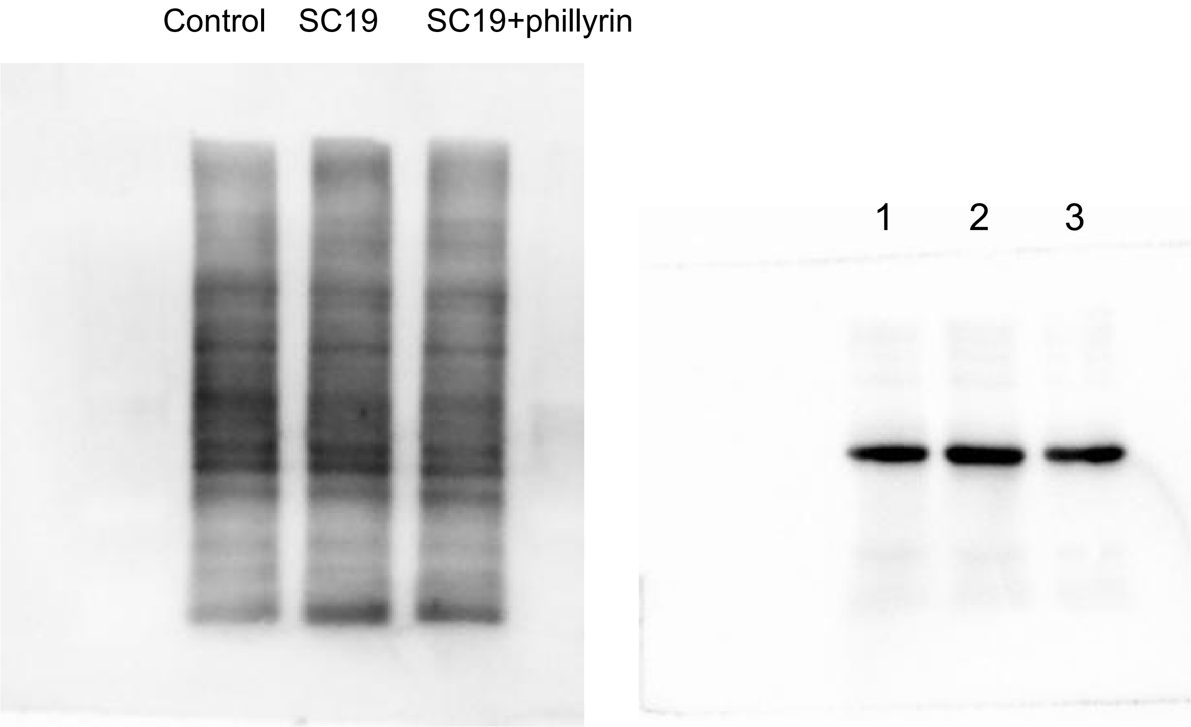

Western blot analysis of phillyrin treated tight junction protein ZO-1 of NPTr cells were infected by SC19 with or without 64μg/mL phillyrin.

Control: ZO-1 of NPTr cells

SC19: ZO-1 of NPTr cells were infected by SC19 without phillyrin.

SC19+phillyrin: ZO-1 of NPTr cells were infected by SC19 with 64μg/mL phillyrin.

1,2,3: β-actin
